# Advanced Structural Health Monitoring Method by Integrated Isogeometric Analysis and Distributed Fiber Optic Sensing

**DOI:** 10.3390/s21175794

**Published:** 2021-08-28

**Authors:** Thein Lin Aung, Ninshu Ma, Kinzo Kishida, Artur Guzik

**Affiliations:** 1 Graduate School of Engineering, Osaka University, 2-1 Yamadaoka, Suita 565-0871, Japan; 2 Joining and Welding Research Institute, Osaka University, 11-1, Mihogaoka, Ibaraki 567-0047, Japan; 3 Neubrex Co., Ltd., 1-1-24 Sakaemachi-dori, Kobe 650-0024, Japan; kishida@neubrex.jp (K.K.); guzik@neubrex.jp (A.G.)

**Keywords:** structural health monitoring, numerical method for remote monitoring, distributed fiber optic sensing, isogeometric analysis

## Abstract

Attempts in digital management of structures are among the most popular topics in the trend of Information of Things (IoT). However, the implementation lags behind. This work recognized that Computer Aided Design (CAD) comprises the core of modern engineering; thus, most digital information can be available if CAD is used not only in design but also for life cycle structural health monitoring (SHM). Based on this concept, the newly designed method utilizes the isogeometric analysis (IGA) tool to include the Distributed Fiber Optic Sensing (DFOS) information by proposing a fiber mesh model. The IGA model can be obtained directly from CAD, and the boundary conditions can be provided directly or indirectly from DFOS in real time and remotely. Hence a practical method of SHM is able to achieve highly efficient and accurate numerical model creation, which can even accommodate non-linear constitutive property of materials. The proposed method was applied to a pipe deformation model as an example. The inverse analysis method is also shown to determine the contact force for loading on the pipe, which shows the potential for many engineering applications.

## 1. Introduction

The intention to develop systems for digital management of engineering structures is clearly realized today, but the practical method is still in a very primitive stage. The primary problems are summarized below:

- Numerical analysis is dominated by FEM, but it does not utilize the CAD model data. Creation of a FEM model needs manual preprocessing jobs, which translates to high costs for general applications in engineering;
- Spatial resolution, accuracy, robustness and installation costs of onsite sensors need to be considered for practical usage. Using multiple discrete sensors such as strain gauges to cover a wide area requires complex wiring and data acquisition instruments with limited scalability.

Distributed Fiber Optic Sensing (DFOS) is a highly developed technology, and recent instruments can measure strain in centimeter spatial resolution with precision below 0.1 microstrain depending on the sensing cable, technique of fiber interrogation and sample averaging counts [1,2,3,4,5]. Fiber optic (FO) cables are small, lightweight, resistant to harsh environments, are installed more and more in the world and are soon to be the only permanent communication infrastructure on this planet. In Japan, any location in the main island is within 3 km distance range to a telecommunications line. Thus, utilizing the public communication fiber cables for monitoring structures such as bridges or dams will not require the purchasing of interrogators, as they may be located in the centers of local stations, especially in the future. Moreover, a fiber optical sensor does not need a power supply and has the ability for self-diagnostics in the case of breakage, which are the key factors of the cost in current systems. DFOS systems have been used in studies for monitoring concrete structures, power plants, aircraft wings and more [6,7,8,9,10,11,12,13,14,15].

**Figure 1:**
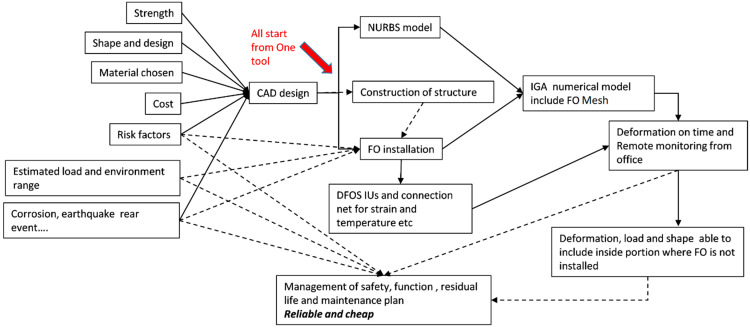
Block diagram of proposed method. The solid lines indicate direct actions, while the dashed lines illustrate the links of solutions to the factors of concern.

In many projects of DOFS that the authors have experienced, CAD was the tool used worldwide among engineers, and even the sensing FO routes are designed within CAD scheme. CAD data are the default standard of tools for communication among different clusters of engineers. The utilization of CAD data resource is an elegant route which results in the focus on the isogeometric analysis (IGA) method. The IGA method utilizes Non-Uniform Rational B-Spline (NURBS) functions which can also be used to make CAD models in principle. Combining IGA model with DFOS technology is the best scheme relative to the authors’ knowledge, and hence this work is initiated. Combining the real-time distributed sensing capabilities of DFOS and high efficiency of IGA, a remote structural monitoring system can be developed as shown schematically in Figure 1.

There are many concerns included in the final CAD design data. The concerns such as mechanical strength, shape of design, material used and cost factors can be called known factors. While the risk of design could be the biggest loading after construction, corrosion, environment conditions and unpredicted events of impact or earthquakes are based on the ‘best effort spirit’. Moreover, the maintenance costs are proved to be higher than initial construction cost and effects, and the costs of maintenance are highly related to the how correctly the structure is deformed and aged.

The physical sensing system is crucial in general but contains many details than only installing a FO sensor. Due to the access conditions and FO bending properties, etc., the FO route cannot be freely designed, and a CAD plan is necessary for the ease of installation. In addition, there is a gap in information between directly measured strains and the necessary engineering data. The measurement data are usually sparse and limited in terms of type of information, demanding the use of numerical analysis and finite element tools in order to translate data into insights [16,17]. For example, DFOS can be used to obtain distributed strain measurements along the optical fiber, and numerical tools are used to estimate deformation, loading conditions and stress distributions in the structure. This job needs to be realized not as the DFOS system but as a more powerful analysis tool. Again, the solution is expected to be IGA.

Isogeometric analysis (IGA) is numerical simulation a tool which recently gained traction because of its superior accuracy and efficiency over traditional FEM [18,19,20,21,22]. As mentioned previously, IGA uses Non-Uniform Rational B-Spline (NURBS) basis/shape functions instead of classical polynomial shape functions to represent geometry and solution fields. NURBS functions are also used in computer-aided design (CAD) systems, which renders the transition from CAD to analysis more streamlined [23,24]. In addition, IGA can utilize exact geometry and higher order continuity than compared to classical finite elements. The convergence properties and efficiency of IGA has been extensively studied, for example, in the references [19,20,25,26,27]. By combining the real-time distributed sensing capabilities of DFOS and high efficiency of IGA, a remote structural monitoring system can be developed as shown in Figure 1.

In this paper, a novel formulation for simulating fiber strains using integrated IGA and DFOS in direct (forward) calculation is introduced as a first step to develop a unified monitoring system. The fiber mesh for DFOS is modelled with NURBS basis functions, which facilitates easy input from CAD to an isogeometric system. Then, the proposed formulation is applied to a numerical example and compared with experiment data. Finally, an inverse method for estimating unknown boundary conditions from observed DFOS strain data is proposed and validated with experiment.

### Distributed Fiber Optical Sensing: State of the Art

Fiber optic sensors are superior over traditional point sensors such as strain gauges because of their ability to perform distributed measurements using a single fiber without the need for multiplexing multiple sensors. There are several types of distributed optical strain sensing technologies, namely fiber Bragg grating (FBG) sensors or backscattering based sensors such as Brillouin, Rayleigh and Raman [1,28,29]. FBG sensors have been widely used in the past research studies [7,8], but backscattering based sensors have recently received attention because of their improved spatial resolution and lateral sensing capabilities [3,6,11,30]. In addition, a backscattering based instrument can utilize an ordinary telecommunication type glass fiber without the need for any modification. Rayleigh Frequency Shift (RFS) principle, also called Tunable Wavelength Coherent Optical Time Domain Reflectometry (TW-COTDR) by Neubrex, is based on Rayleigh backscattering phenomenon, which consists of elastic scattering. When light passes through an optical fiber, random refractive index fluctuations along the fiber causes backscattering [5]. This can be presented as the random distribution of the power spectrum in TW-COTDR. The presence of a variation in strain and/or temperature causes a shift in the Rayleigh frequency. The magnitude of the desired sensing variables (strain/temperature) can be obtained by comparing the measured frequency differences between the reference state and testing state via cross-correlation. The spatial resolution and accuracy of backscattering sensing depend on the sliding window length of the frequency demodulation.

The DFOS instrument used for the experiments in this paper (Neubrescope, NBX7031, Neubrex Co. Ltd., kobe, Japan) primarily supports Pre-Pump Pulse Brillouin Optical Time-domain Analysis (PPP-BOTDA) and TW-COTDR techniques for temperature compensated strain measurements [2,3]. TW-COTDR technique was used in this study for its greater stability in high spatial resolution settings. A thorough explanation on the working principles of PPP-BOTDA and TW-COTDR can be found in the relevant references [4,31,32].

## 2. Formulation of Integrated IGA and DFOS

### 2.1. Coordinate Systems

**Figure 2:**
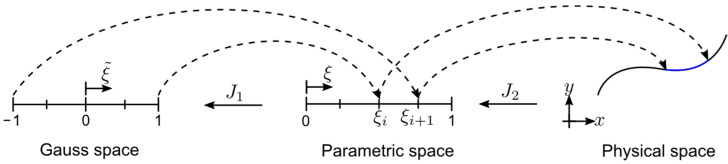
Coordinate systems and mappings.

In isoparametric formulations such as IGA, there are primarily three coordinate systems (spaces) as illustrated by a univariate example in Figure 2. The first one is Gauss space, where the Gauss integration points are prior defined. The Gauss space spans from −1 to 1. The second space is the parametric space where the basis functions are evaluated. In IGA terminology, it also represents the parent element and normalized knot vector of the NURBS geometry [18]. The last space is the physical coordinate system in which the geometry itself exists.

The coordinate transformation from Gauss space to parametric space follows a linear mapping:

(1) $$\xi =\frac{1}{2}\left[\left(\xi_{i+1}-\xi_{i}\right)\tilde{\xi }+\left(\xi_{i+1}+\xi_{i}\right)\right].$$

where $\left\{\xi_{i},\xi_{i+1}\right\}$ is known as a *knot span* and the Jacobian $J_{1}$ from parametric space to Gauss space is the following.

(2) $$J_{1}=\frac{\partial \xi }{\partial \tilde{\xi }}=\frac{1}{2}\left(\xi_{i+1}-\xi_{i}\right).$$

A parametric curve c(ξ) is defined by the product of univariate basis functions $N_{i}$ and control point coordinates C of the following.

(3) $$c\left(\xi \right)=\overset{n}{\underset{i=1}{\sum }}N_{i}\left(\xi \right)\;C_{i},\;\;\xi \in \mathbb{R};\;c,C_{i}\in {\mathbb{R}}^{3}.$$

In isoparametric formulation, the same basis can be used to represent fields such as displacement or strain. In that case, the control point coordinates C are replaced by *control parameters*. For instance, the displacement field is expressed as follows.

(4) $$u\left(\xi \right)=\overset{n}{\underset{i=1}{\sum }}N_{i}\left(\xi \right)\;d_{i},\;\;\xi \in \mathbb{R};\;u,d_{i}\in {\mathbb{R}}^{3},$$

The Jacobian from physical to parametric space can be derived as follows.

(5) $$J_{2}=\frac{\partial x}{\partial \xi }={\left[\begin{array}{ccc} \frac{\partial x}{\partial \xi } & \frac{\partial y}{\partial \xi } & \frac{\partial z}{\partial \xi } \end{array}\right]}^{T},$$

(6) $$\left|J_{2}\right|=\sqrt{{\left(\frac{\partial x}{\partial \xi }\right)}^{2}+{\left(\frac{\partial y}{\partial \xi }\right)}^{2}+{\left(\frac{\partial z}{\partial \xi }\right)}^{2}},$$

(7) $$\frac{\partial x}{\partial \xi }=\overset{n}{\underset{i=1}{\sum }}\frac{\partial N_{i}}{\partial \xi }C_{xi},\;\frac{\partial y}{\partial \xi }=\overset{n}{\underset{i=1}{\sum }}\frac{\partial N_{i}}{\partial \xi }C_{yi},\;\;\frac{\partial z}{\partial \xi }=\overset{n}{\underset{i=1}{\sum }}\frac{\partial N_{i}}{\partial \xi }C_{zi}.$$

### 2.2. B-Spline and NURBS Basis Functions

Basis functions are a crucial part of isoparametric formulation and isogeometric analysis since they are used to represent both geometry and solution fields. Univariate B-Spline basis functions of order *p* are defined by the *Cox–de Boor* recursive formula [33,34]:

(8) $$N_{i,0}\left(\xi \right)=\{\begin{array}{l} 1\;\;,\;\mathrm{if}\;\xi_{i}\leq \xi <\xi_{i+1}\\ 0\;\;,\;\mathrm{otherwise} \end{array}\mathrm{and}\;N_{i,p}\left(\xi \right)=\frac{\xi -\xi_{i}}{\xi_{i+p}-\xi_{i}}N_{i,p-1}\left(\xi \right)+\frac{\xi_{i+p+1}-\xi }{\xi_{i+p+1}-\xi_{i+1}}N_{i+1,p-1}\left(\xi \right),$$

where ξ is the parameter and $\xi_{i}$ denotes ith knot value in the knot vector $\Xi =\left\{\xi_{1},\xi_{2},\ldots ,\xi_{n+p+1}\right\}$. A knot vector is a non-decreasing set of real numbers in the parametric space. In this paper, it always refers to the *open* knot vector where the first and last entries are repeated p+1 times. This ensures that the first and last control points interpolate (coincide) with the physical geometry. Moreover, we will always use normalized knots between 0 and 1.

Non-Uniform Rational B-Spline (NURBS) basis functions are formed by projected transformation of B-Splines to allow for exact representation of conic sections. Univariate NURBS basis is defined as follows:

(9) $$R_{i}^{p}\left(\xi \right)=\frac{N_{i,p}\left(\xi \right)w_{i}}{\sum_{i=1}^{n}N_{i,p}\left(\xi \right)w_{i}},$$

where $w_{i}$ is a weight vector. Trivariate NURBS basis functions are defined as the tensor product of univariate B-Spline basis functions and respective weights as follows.

(10) $$R_{i,j,k}^{p,q,r}\left(\xi ,\eta ,\zeta \right)=\frac{N_{i,p}\left(\xi \right)M_{j,q}\left(\eta \right)L_{k,r}\left(\zeta \right)w_{i,j,k}}{\sum_{i=1}^{n}\sum_{j=1}^{m}\sum_{k=1}^{l}N_{i,p}\left(\xi \right)M_{j,q}\left(\eta \right)L_{k,r}\left(\zeta \right)w_{i,j,k}}.$$

In practice, the weight values are assigned to the control points and invoked during the evaluation of basis functions. The first derivatives of B-Spline basis functions are defined by the following.

(11) $$\frac{d}{d\xi }N_{i,p}\left(\xi \right)=\frac{p}{\xi_{i+p}-\xi_{i}}N_{i,p-1}\left(\xi \right)-\frac{p}{\xi_{i+p+1}-\xi_{i+1}}N_{i+1,p-1}\left(\xi \right),$$

The first derivatives of NURBS basis functions are defined by the following.

(12) $$\frac{d}{d\xi }R_{i,p}\left(\xi \right)=w_{i}\frac{\frac{d}{d\xi }N_{i,p}\left(\xi \right).\sum_{j=1}^{n}N_{j,p}\left(\xi \right)w_{j}-N_{i,p}\left(\xi \right).\sum_{j=1}^{n}\frac{d}{d\xi }N_{j,p}\left(\xi \right)w_{j}}{{\left[\sum_{j=1}^{n}N_{j,p}\left(\xi \right)w_{j}\right]}^{2}}.$$

The derivatives with respect to physical coordinates can be obtained by using the following Jacobian.

(13) $$\frac{dN\left(\xi \right)}{dx}=\frac{dN\left(\xi \right)}{d\xi }\frac{d\xi }{dx}=\frac{dN\left(\xi \right)}{d\xi }\left|J_{2}^{-1}\right|.$$

In summary, orders of basis functions, knot vectors, control point coordinates and weight vectors are necessary to define a NURBS geometry and its derivatives. NURBS basis functions have the properties of the following [19,33]:

- Non-negativity;
- Partition of unity, which means basis functions add up to 1;
- Locality, which means the support or influence of a basis function extends only a limited region within the geometry;
- Convex hull, which means the geometry always lie inside the control polygon;
- Affine covariance, which means the geometry transforms in the same manner as the control points.

### 2.3. Isogeometric Analysis

The implicit elastic IGA is similar to that of traditional FEM except basis functions and connectivities [20], and the use of control points instead of node points. The linear IGA formulation of solid mechanics is briefly reviewed in this section.

Using the virtual work principle, the potential energy in the domain Ω can be derived as follows:

(14) $$\Pi =\int_{\Omega }\varepsilon^{T}\sigma d\Omega -\int_{\Omega }u^{T}bd\Omega -\int_{\Omega }u^{T}td\Omega ,$$

where b is body force and t is surface traction force. After discretization of the domain Ω by the shape functions $N_{i}$, the strain distribution can be obtained from the strain-displacement relation, which in turn can be derived from the derivative of displacement field:

(15) ε=Bd,

(16) $$B=\left[\begin{array}{ccccccc} N_{1,x} & 0 & 0 & \cdots & N_{n,x} & 0 & 0\\ 0 & N_{1,y} & 0 & \cdots & 0 & N_{n,y} & 0\\ 0 & 0 & N_{1,z} & \cdots & 0 & 0 & N_{n,z}\\ N_{1,y} & N_{1,x} & 0 & \cdots & N_{n,y} & N_{n,x} & 0\\ 0 & N_{1,z} & N_{1,y} & \cdots & 0 & N_{n,z} & N_{n,y}\\ N_{1,z} & 0 & N_{1,x} & \cdots & N_{n,z} & 0 & N_{n,x} \end{array}\right],$$

where $N_{i,x}\equiv \frac{\partial N_{i}\left(\xi \right)}{\partial x}$ and B is the strain-displacement matrix.

Stress can be derived from strain and constitutive relation in terms of the material matrix D.

(17) σ=Dε.

Then, the potential energy can be approximated as follows.

(18) $$\Pi =\overset{n_{e}}{\underset{e=1}{\sum }}\left[\int_{\Omega_{e}}B^{T}d^{T}DBd{d\Omega }_{e}-\int_{\Omega_{e}}N^{T}d^{T}b{d\Omega }_{e}-\int_{\Gamma_{e}}N^{T}d^{T}td\Gamma_{e}\;\right].$$

Using the derivatives with respect to discretized displacements, the stationary condition for minimum energy can be obtained as follows.

(19) $$\frac{\partial \Pi }{\partial d}=0,\;$$

(20) $$\overset{n_{e}}{\underset{e=1}{\sum }}\left[\int_{\Omega_{e}}B^{T}DBd{d\Omega }_{e}-\int_{\Omega_{e}}N^{T}b{d\Omega }_{e}-\int_{\Gamma_{e}}N^{T}td\Gamma_{e}\;\right]=0.$$

Finally, the element stiffness matrix and force vectors can be written as follows.

(21) $$K_{e}=\int_{\Omega_{e}}B^{T}DB{d\Omega }_{e},\;$$

(22) $$f_{e}=\int_{\Omega_{e}}N^{T}b{d\Omega }_{e}+\int_{\Gamma_{e}}N^{T}td\Gamma_{e}.$$

After assembling the global stiffness matrix and force matrix, discretized displacements at the control points can be solved with a system of linear simultaneous equations.

(23) $$K_{e}\Rightarrow K,\;f_{e}\Rightarrow f,\;$$

(24) Kd=f,

The strain and stress distributions can be computed from Equations (15) and (17).

### 2.4. Fiber Mesh

The geometry of the optical fiber can be represented as a univariate parametric NURBS curve in 3D space by assuming that the mass and stiffness of the optical fiber have negligible contributions to the structural system. Setting the parametric fiber coordinates as ξ, the physical coordinates x can be computed from the following:

(25) $$x\left(\xi \right)=\overset{n}{\underset{i=1}{\sum }}N_{i}\left(\xi \right)C_{i},\;$$

and the 1D physical fiber coordinate s is equivalent to the arclength of the curve, which can be found as follows.

(26) $$s\left(\xi \right)=\int_{0}^{s}ds=\int_{0}^{\xi }\left|J_{2}\right|\;d\xi .$$

The transformation from physical to parametric fiber coordinates can be performed by using an iterative method such as a golden section search.

In the calculation of fiber strains described in the following section, tangent vector at the sampling point on fiber is required. The unit tangent vector of the fiber at parameter ξ can be derived as follows.

(27) $$\tilde{t}\left(\xi \right)=\frac{dx\left(\xi \right)}{d\xi }=\overset{n}{\underset{i=1}{\sum }}\frac{dN_{i}\left(\xi \right)}{d\xi }C_{i},\;$$

(28) $$t\left(\xi \right)=\frac{\tilde{t}\left(\xi \right)}{||\tilde{t}(\xi )||}.$$

**Figure 3:**
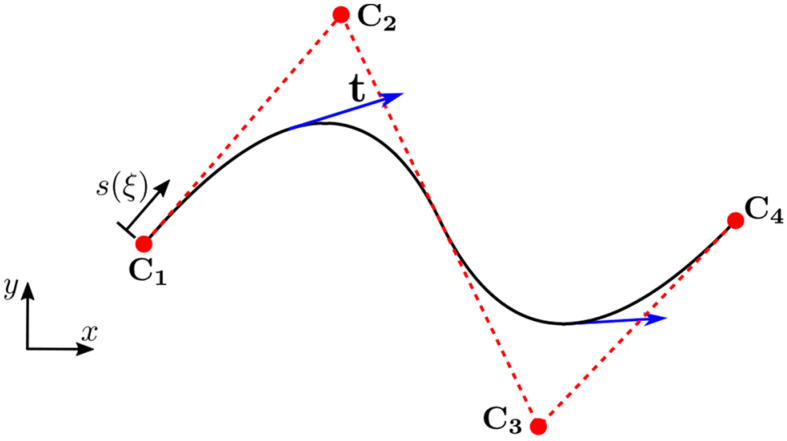
A NURBS curve showing control points ($C_{1},C_{2},C_{3},C_{4}$) and tangent vectors t. p=2; Ξ={0,0,0,0.5,1,1,1}; all weights are equal to 1.

An example of a second order two-dimensional NURBS curve and tangent vectors are illustrated in Figure 3.

### 2.5. Conversion from Strain Tensor to Fiber Strains

After the systems of equations in Equation (24) are solved, the strain distribution can be obtained from Equation (15). In order to calculate one-dimensional fiber strains, the strain tensor on the surface of the structure where the fiber is attached can be used along with the tangent vector t of the fiber at the sampling point.

The one-dimensional fiber strains at a sampling point s, which corresponds to global coordinates x, can be computed by projecting the strain tensor on the tangential direction of the fiber.

(29) $$\bar{\varepsilon_{f}}\left(s\right)=t^{T}\varepsilon \;t\;,\;$$

(30) $$\varepsilon \left(x\left(s\right)\right)=\left[\begin{array}{ccc} \varepsilon_{xx} & \varepsilon_{xy} & \varepsilon_{xz}\\ \varepsilon_{yx} & \varepsilon_{yy} & \varepsilon_{yz}\\ \varepsilon_{zx} & \varepsilon_{zy} & \varepsilon_{zz} \end{array}\right],\;t\left(s\right)=\left(\begin{array}{c} t_{x}\\ t_{y}\\ t_{z} \end{array}\right).$$

**Figure 4:**
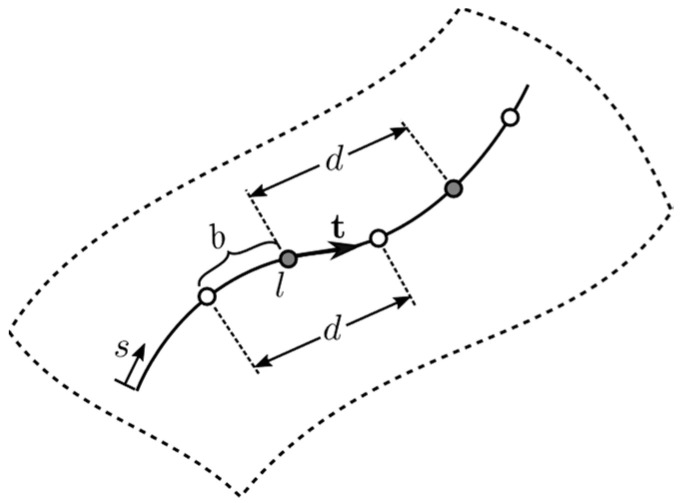
Illustration of fiber strain sampling points (circles) with sampling interval b and sampling resolution d; sampling resolution is numerically the same as the moving average window.

In practice, fiber strains are not measured as single point strains but averaged over a distance *d* as illustrated in Figure 4. Hence, the moving average fiber strain over length d at sampling location l can be expressed as the following.

(31) $$\varepsilon_{f}\left(l\right)=\frac{1}{d}\int_{l-\frac{d}{2}}^{l+\frac{d}{2}}\bar{\varepsilon_{f}}\left(s\right)ds,\;$$

(32) $$\varepsilon_{f}\left(l\right)=\frac{1}{d}\overset{nipt}{\underset{i=1}{\sum }}\bar{\varepsilon_{f}}\left(s_{i}\right)\left|J_{1i}\right|\left|J_{2i}\right|w_{i}.$$

**Figure 5:**
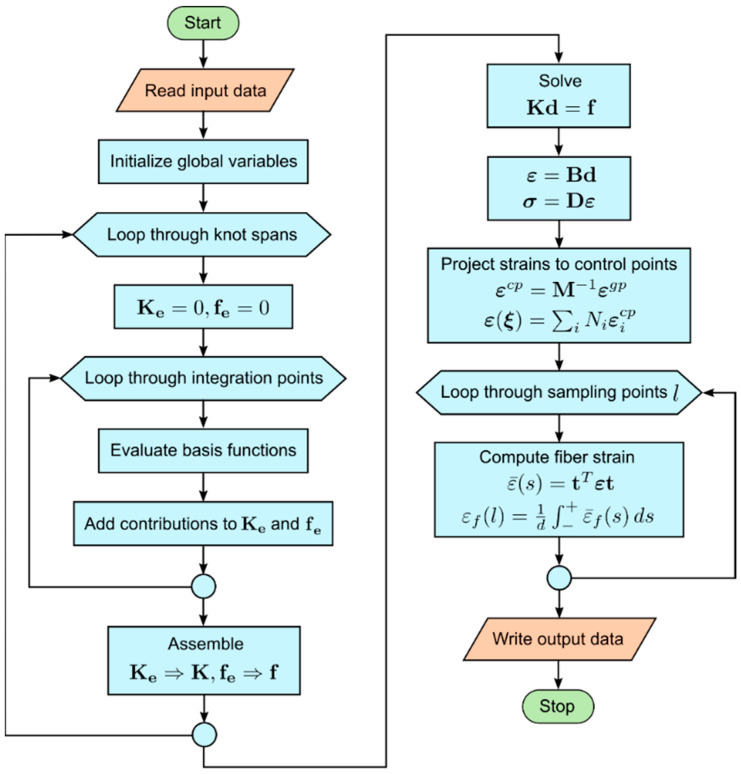
Flow chart for IGA elastic stress analysis and calculation of fiber strains.

A flow chart, which summarizes the elastic stress analysis and fiber strain calculation at all sampling points, is shown in Figure 5.

### 2.6. Merits of IGA and DFOS

The primary advantages of the proposed formulation using IGA and DFOS compared to traditional FEM and point strain gauges are outlined in Table 1. IGA allows the use of CAD data for the structure and optical fiber in the form of NURBS control points and knot vectors for a streamlined Computer-Aided Engineering (CAE) workflow. The existing concise geometric algorithms can also be implemented in the analysis code for efficient and accurate computations. Moreover, the abilities to represent exact geometry regardless of the analysis mesh size and high efficiency in terms of total degree-of-freedom (DOF) in IGA have been verified by numerous researchers [18,22].

Comparison of DOFS and traditional strain gauges reveals that DFOS can provide distributed strain data using a simple setup and possesses high resolution, long range and remote monitoring capabilities. Among several types of distributed optical strain sensors such as fiber Bragg grating (FBG) or Rayleigh backscattering sensors (RBS) [1], backscattering based sensors have higher spatial resolution than FBG sensors [30]. The proposed fiber mesh is independent of the type of fiber strain sensor since only the geometry of the fiber and measurement information is required as inputs. Using the fiber mesh and upcoming inverse analysis method, real time structural state such as deformation, loading, stress and strain distributions can be inferred from the observed fiber strain data.

## 3. Stress-Strain Analysis with Integrated IGA and DFOS

In this section, an application example of the proposed formulation using integrated IGA and DFOS for elastic stress-strain analysis is presented with an experimental model of a test pipe and DOFS for strain sensing. IGA and the corresponding DFOS fiber mesh equations were implemented in the authors’ in-house code, JWRIAN-IGA.

### 3.1. Experiment with a Cylindrical Pipe

#### 3.1.1. Experiment Setup

**Figure 6:**
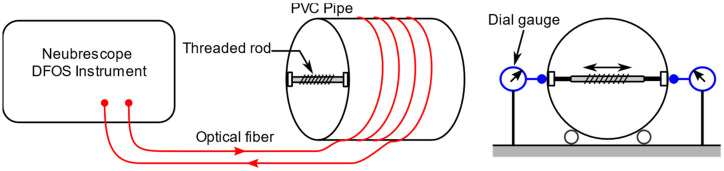
Schematics of experiment setup.

**Figure 7:**
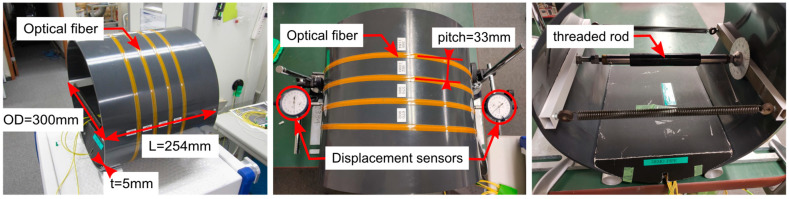
Pipe dimensions and experiment setup showing optical fiber, displacement sensors and threaded rod.

The schematic of the experiment setup is illustrated in Figure 6, and actual model is shown in Figure 7. The pipe was made of polyvinyl chloride (PVC) material with an outer diameter of 300 mm and thickness of 5 mm. The material properties of the pipe are shown in Table 2. The optical fiber was wounded four loops around the outer surface of the pipe with a pitch of 33 mm, and a protective tape was applied on top of the fiber. The fiber coordinates on the pipe surface were marked for later use in the simulation. The loading (displacement) was applied by turning a threaded rod (screw) at the mid-section of the pipe. The load was distributed along the length of the pipe by a pair of rectangular beams. The total applied displacement was measured with two dial gauges on opposite sides across the diameter of the pipe.

DFOS strain data were measured by a Neubrescope (NBX7031) instrument using Rayleigh backscattering sensor (RBS) because of its excellent accuracy and spatial resolution. Specifically, the Tunable Wavelength Coherent Optical Time Domain Reflectometry (TW-COTDR) mode was used to measure fiber strains in 1 cm intervals.

#### 3.1.2. Experiment Procedure

The experiment was performed for five cases of applied displacements named M1 to M5 to check the response of the fiber strains with respect to deformation of the pipe. The experiment parameters, such as fiber strain sampling interval, are shown in Table 3. The spatial resolution is defined by the moving average distance of the fiber strain measurements.

Before the experiment, a calibration reading was performed to obtain a reference strain distribution when the pipe was under minimal deformation. Then, a certain amount of displacement was incrementally applied by turning the threaded rod. The net prescribed displacement was calculated as the average of the two dial gauge readings. The displacement caused deformation of the pipe in an oval shape and it was detected by the optical fiber as 1D fiber strains. Then, the fiber strain readings were performed and the measured fiber strains were adjusted by subtracting the reference to obtain the net fiber strains. After the fiber strains are recorded, the stroke was increased, and the process was repeated in order to obtain the fiber strains for all load cases.

### 3.2. Fiber Strain Measurements

**Figure 8:**
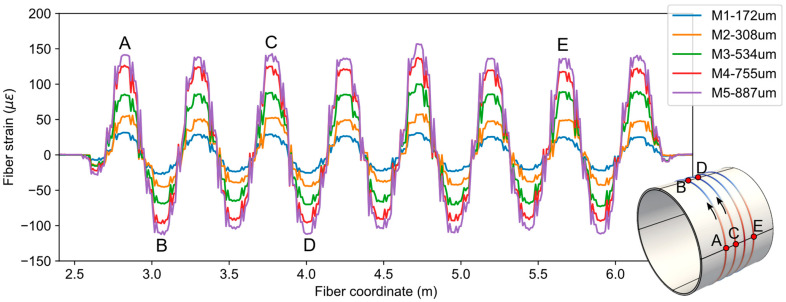
Fiber strain measurement data from experiments.

The resulting net fiber strains for each load case (M1 to M5) along with average displacement values are shown in Figure 8. As expected, the fiber strains oscillate between positive (tensile) and negative (compressive) values because of the pipe’s oval shaped mode of deformation. Specifically, the part of fiber on the left and right regions of the pipe experienced tensile strains, and the fiber on the top and bottom regions experienced compressive strains.

**Figure 9:**
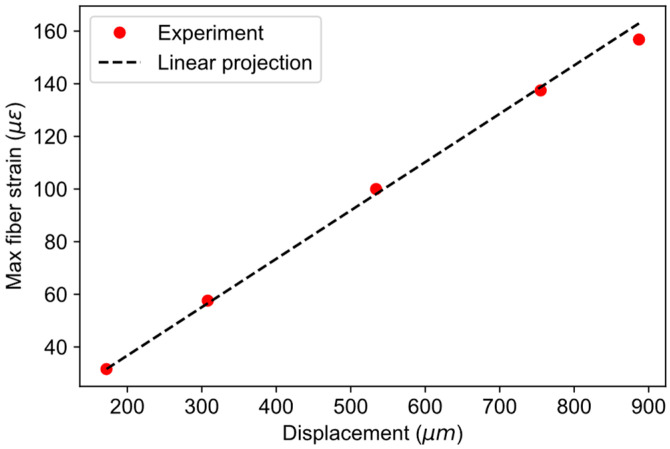
Response of fiber strains on applied displacement to the pipe.

The response of the fiber strains with respect to the magnitude of pipe deformation can be checked by plotting the value of maximum fiber strains vs. applied displacement values. Such a plot is shown in Figure 9, along with the linear projection using the first measurement point. It can be observed from the plot that the fiber strains follow linear projection very well, justifying the use of a linear model in the simulations.

### 3.3. Forward IGA Using Idealized Boundary Conditions

In this section, the pipe and fiber models are simulated with forward IGA using simplified boundary conditions. The calculation results are then compared with experiment data.

#### 3.3.1. Geometry and Analysis Mesh

**Figure 10:**
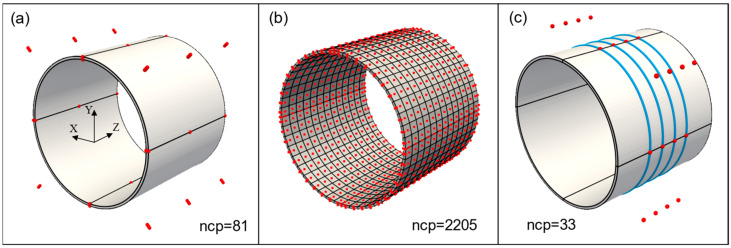
(**a**) Pipe geometry and control points (red). Black lines show C^0^ continuity; (**b**) Analysis mesh and control points after h-refinement; (**c**) fiber geometry (blue) and fiber control points (red).

The NURBS geometry of the test pipe was created using trivariate second order functions and 81 control points. The resulting geometry is an exact representation of a hollow cylinder. Due to the tensor product structure of NURBS, it can be imagined as bending a plate that joins the end surfaces in order to achieve a cylindrical shape. This standard construction also introduces three C^0^ continuous lines along the circumference, as shown in Figure 10a. The basis functions are C^1^ continuous everywhere else.

Although the geometry in Figure 10a is analysis suitable, h-refinement using knot insertion [35] was performed to create a finer mesh and to improve analysis accuracy. The refined mesh and final control points are shown in Figure 10b. Note that the refinement does not modify the geometry, and the refined IGA mesh has a smaller number of total DOF than compared to traditional FEM mesh of the same size. The refined analysis mesh consists of 611 knot spans (elements) and 2205 control points. Each control point has three translational DOF, producing a total of 6615 DOF. The accompanying fiber geometry was created by using univariate second order functions and 33 control points, and the resulting helical curve is shown in Figure 10c.

#### 3.3.2. Boundary Conditions

Generally, the control points of a NURBS geometry are not interpolatory, meaning the displacements of control points are different from actual displacements of the geometry. The required control point displacements for a given displacement distribution can be derived by using interpolation or fitting methods [22]. However, the pipe model in Figure 10a has C^0^ continuous areas that are interpolatory and displacement can be directly applied in those areas.

**Figure 11:**
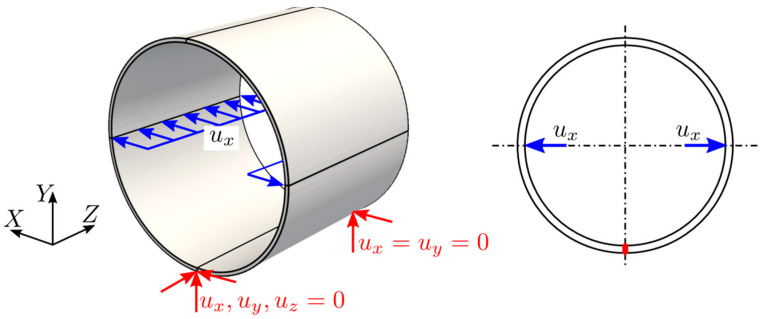
Idealized Boundary conditions.

For the simulation of the pipe model, a uniform displacement was applied along the inner surface of the pipe, as shown in Figure 11. The displacement value was directly applied to the control points since the geometry was interpolatory at those locations. The rigid body motion was constrained along the bottom edge by using multi-point constraints.

### 3.4. Results and Discussion

**Figure 12:**
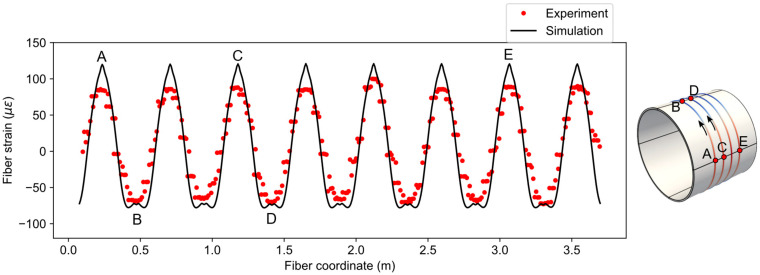
Comparison of fiber strains between simulation and experiment for u=534 μm.

The fiber strain distribution from the simulation is compared with experiment data in Figure 12 for load case three (u=534 μm). The simulated fiber strains have the moving average window of 2 cm, which is numerically the same as the spatial resolution. Moreover, the fiber coordinates of the experiment data have been offset to match the simulation. From Figure 12, it can be observed that the trend of the simulated data closely follows the experiment but the peak values of tensile strains in the simulation are higher than the experiment. The reason for this discrepancy is the idealized concentrated loading described in the previous section. The concentrated loading caused a sharp peak in the strain distribution, which consequently affected the one-dimensional fiber strain. In addition, the loading may not have been uniform along the length of the pipe, as is assumed in the simulation.

**Figure 13:**
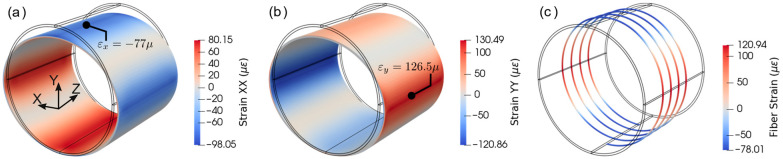
Normal strains distributions: (**a**) strain-XX and (**b**) strain-YY; (**c**) simulated fiber strain distribution.

In order to verify how the fiber strains correlate with surface strains in the tangential direction, normal strains on the pipe surface can be compared with fiber strains at the same location. It can be observed from Figure 13 that the fiber strains have strong correlation with strain-XX and YY in principal locations shown in Figure 13a,b, where the tangent of the fiber is nearly parallel to the coordinate axes. However, they are not identical because the fiber was not perfectly parallel to the axes and moving average effect that was considered.

**Figure 14:**
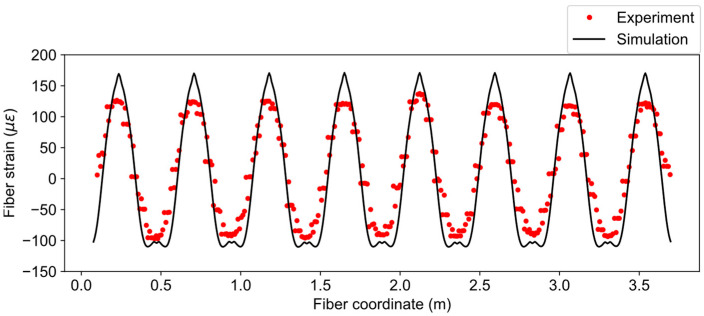
Comparison of fiber strains between simulation and experiment for u=755 μm.

The fiber strain results for the applied displacement of 755 μm (case 4) are compared in Figure 14. The same phenomenon (similar trend but sharp peak) can also be observed for this case, although the magnitudes of the fiber strains are higher than the previous case.

## 4. Estimation of Boundary Conditions Using Inverse IGA

### 4.1. Concept of Inverse IGA

The results in the previous section showed that the simulation overpredicted fiber strains in the tensile side. The discrepancies can be attributed to different boundary conditions between experiment and simulation. This situation is not isolated to the current case; the boundary conditions are often unknown in real-world scenarios. However, the loading force is an important parameter for determining structural state and safety. Hence, a numerical method for estimating boundary conditions from the available fiber strain data is fruitful.

The boundary conditions can be estimated by minimizing the error between observed fiber strains and simulated fiber strains using an optimization approach. This estimation of unknown variables (boundary conditions) from known experiment data (fiber strains) is termed as *inverse* IGA. The estimated boundary conditions can then be used in forward simulation to obtain displacement and stress distributions. It should be noted that an exact inverse is not possible since the fiber strain calculation using Equation (29) is irreversible. Previous studies have proposed the inverse method using least squares for plates [16], using inverse FEM (iFEM) for shells and plates [17] and, recently, using inverse IGA-iFEM for shells with strain gauge rosettes [36]. However, our formulations of integrated IGA and DFOS presented in Section 2 and the following inverse method are substantially different since our method explicitly considers the geometry of the fiber and does not impose any restriction on the location or resolution of sampling points.

### 4.2. Estimation of Boundary Conditions for the Pipe Model

**Figure 15:**
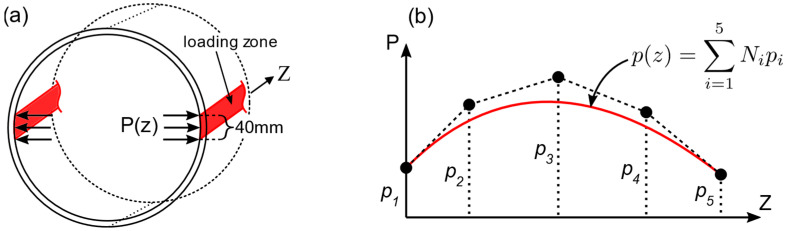
(**a**) Pressure application and loading zone; (**b**) Parametrization of the pressure distribution function along Z-direction; $p_{i}$ are free parameters which influence the shape of p(z).

In this section, the proposed method of inverse IGA is described by using the aforementioned pipe model as an illustrative example. The unknown loading along the pipe was defined as a pressure distribution on the surface area of the pipe that was in contact with the rectangular bars (Figure 15a). The variation of pressure along the length (Z) direction of the pipe was represented with a second order B-spline function with five coefficients as free parameters, as shown in Figure 15b.

In order to fit the fiber strains using optimization, the objective function E was defined as the sum of squared errors between observed fiber strains $\varepsilon_{f}^{*}$ and calculated fiber strains $\varepsilon_{f}$ at each sampling point along the fiber.

(33) $$\underset{p_{i}\in {\mathbb{R}}^{5}}{\min}E=\overset{n_{s}}{\underset{i=1}{\sum }}{\left(\varepsilon_{fi}^{*}-\varepsilon_{fi}\right)}^{2}.$$

Here, $\varepsilon_{f}$ refers to the moving average fiber strains as defined in Equation (31). The objective E was minimized with respect to the optimization parameters $p_{i}$ in the pressure distribution function in Figure 15b in order to obtain the best-fit solution in least squares sense. After the pressure distribution was assumed, the analysis followed the same process as the forward calculation to compute simulated fiber strains and least squares objective.

The minimization problem was solved by a numerical optimization method, namely sequential least squares programming (SLSQP). It is a gradient based quadratic optimization method suitable for a small number of parameters and for fairly simple functions without multiple local minimums. The gradients of the objective were evaluated by numerical finite difference method. The termination criterion for the optimization was $\Delta E<10^{-4}\;\mu \varepsilon$ between successive iterations.

### 4.3. Results and Discussion

**Figure 16:**
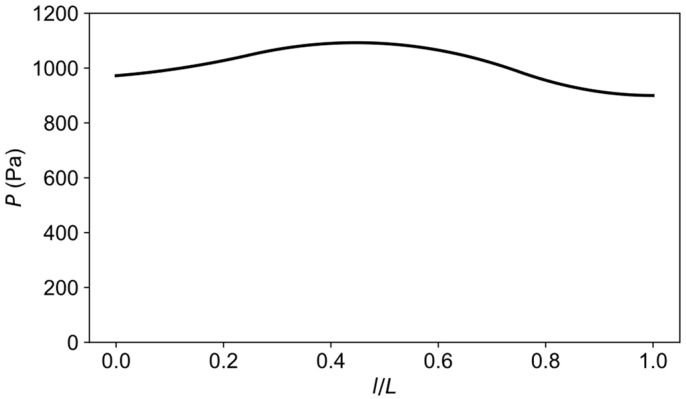
Optimized pressure distribution along the longitudinal direction of the pipe.

**Figure 17:**
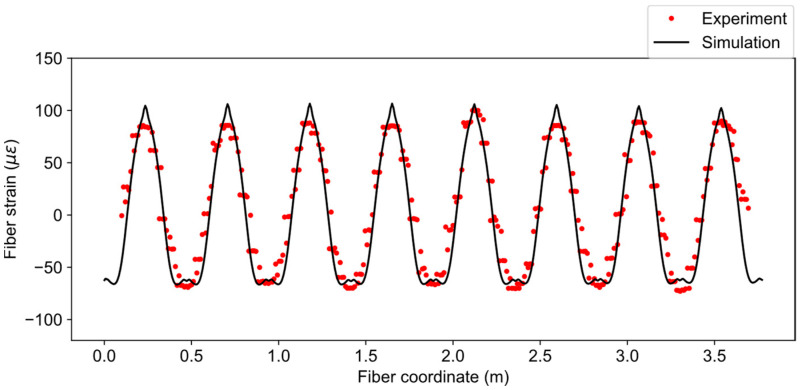
Fiber strain distribution from inverse calculation.

The optimization with input fiber strains from load case three converged after the fifth iteration with a total of 31 objective evaluations including finite difference calculations. The optimized pressure distributions and the fiber strains are shown in Figure 16 and Figure 17, respectively. From this inverse calculation, the pressure was found to be higher in the middle part of the pipe, as is evident from Figure 16. It can be easily understood that the bending of the rectangular beams in the experiment could give rise to a nonuniform loading along the pipe. Table 4 shows the statistics of RMS and maximum errors of the forward and inverse calculations. The boundary conditions from the inverse calculation had lower fiber strain errors than the idealized ones in the forward simulation. The average displacement error between inverse calculation and ground truth was less than 4%, which proves the effectiveness of proposed inverse IGA method for estimating boundary conditions.

The parametrization of boundary condition (pressure distribution) shown in Figure 15b is not unique since a different number of coefficients can be used for the B-spline function. The parametrization influences the shape of possible loading distributions and, hence, should be chosen carefully by considering practicality and computation time. For the current model, it can be deduced that five coefficients were sufficient to reproduce the optimal loading distribution in Figure 16 since more complex shapes do not improve the solution but, instead, increase the number of iterations for convergence.

## 5. Conclusions

In this paper, a novel formulation of integrated IGA and DFOS was proposed and validated by using experiment data. In particular, the newly developed fiber mesh model combines the geometric accuracy of IGA and versatility of DFOS into a single package. Once implemented, the proposed technique can be applied to virtually unlimited structures/geometries as long as the CAD models are available. Hence, this work shows the potential for an efficient Computer-Aided Engineering workflow in the structural monitoring field.

We have developed an in-house code to verify the robustness of the formulation. The continuous fiber strain distribution was computed in a unified isogeometric system using the integrated method. The case study showed that the simulated fiber strains using the assumed boundary conditions in the forward analysis matches the experiment with a few discrepancies.

Another important application of IGA-DFOS model was illustrated as the inverse IGA method in which unknown boundary conditions were estimated from measured fiber strain data. The example problem used parametric optimization to obtain pressure distribution function, which fits the measured fiber strains in least squares sense. The results showed that the inverse IGA method can be used to estimate structural conditions from the observed fiber strains.

## Figures and Tables

**Table 1:** Comparison of proposed technologies with traditional technologies.

IGA	Classical FEM	DFOS	Strain Gauge
Can use CAD model directly	Cannot use CAD model directly	Distributed measurement data	Discrete sensors for multiple locations
Exact geometry	Approximate geometry	High spatial resolution	Single point
Same basis functions for the geometry, fiber model and solution fields	Different basis functions (need data conversion between models)	Long range (~25 km) in a single fiber	Require multiplexers
High efficiency in terms of total DOF in the system	Low efficiency in terms of total DOF in system	Remote, 24/7 monitoring	
$C^{\left(order-1\right)}$ continuous across element boundaries	$C^{0}\;$ continuous across element boundaries	Stable in harsh environments	

**Table 2:** Material properties of the pipe model.

Material Type	Polyvinylchloride (PVC)
Young’s modulus (nominal)	2.8 GPa
Poisson’s ratio (nominal)	0.38

**Table 3:** Measurement parameters in the experiment.

Optical Technology	TW-COTDR
Sampling interval	1 cm
Spatial resolution	2 cm
Averaging count	2^16^
Repeatability	±1 με

**Table 4:** Fiber strain error statistics of uniform loading and optimized loading (inverse calculation); * average displacement.

	Initial Loading	Optimized Loading
RMS fiber strain error	13.421 με (7.318%)	5.863 με (3.197%)
Max fiber strain error	30.539 με (16.652%)	16.245 με (8.858%)
Displacement error	-	3.914% *

## Data Availability

The data presented in this study are openly available in Zenodo at https://doi.org/10.5281/zenodo.5290353, accessed on 24 August 2021.
